# Decoupling Reversible Interface Trapping and Irreversible Bulk Transitions in Solution-Processed Indium Zinc Oxide Thin-Film Transistors

**DOI:** 10.3390/nano16140877

**Published:** 2026-07-16

**Authors:** Dongwook Kim, Hyunji Shin, Hyeonju Lee, Youngjun Yun, Jin-Hyuk Bae, Jaehoon Park

**Affiliations:** 1 School of Semiconductor and Display Technology, Hallym University, Chun-Choen 24252, Republic of Korea; d.kim@hallym.ac.kr (D.K.); hjlee@hallym.ac.kr (H.L.); youngjun.yun@hallym.ac.kr (Y.Y.); 2 School of Electrical and Computer Engineering, University of Seoul, Seoul 02504, Republic of Korea; hshin09@uos.ac.kr; 3 School of Electronics Engineering, Kyungpook National University, Daegu 41566, Republic of Korea; jhbae@ee.knu.ac.kr; 4 School of Electronic and Electrical Engineering, Kyungpook National University, Daegu 41566, Republic of Korea

**Keywords:** reversible recombination, irreversible bulk trapping, indium zinc oxide, thin-film transistor, density of state–energy band alignment

## Abstract

In this study, we systematically decoupled reversible charge transitions via recombination and irreversible bulk trapping via ionization in solution-processed indium zinc oxide thin-film transistors (TFTs) under positive- and negative-bias-stress (PBS and NBS) conditions. We defined highly decoupled degradation behavior by completely evaluating time-dependent transfer characteristics and saturation leakage currents across a range of indium molarities (0.0125 M to 0.2 M). Results indicate that PBS-induced instability is likely governed by a reversible electrostatic neutralization process reducing total effective shallow and deep acceptor-like states, which are dynamically counteracted by interfacial recombination at the dielectric/semiconductor boundary. Conversely, severe degradation under NBS originated from irreversible bulk trapping triggered by the ionization of donor-like oxygen vacancies in a ZnO amorphous random network. Total effective trapped charges were calculated from threshold voltage shifts to clarify these defect kinetics quantitatively; these calculations demonstrated direct correlation with the integrated theoretical capacities of the deep and shallow acceptor-like gap-state distributions. Finally, we propose a comprehensive density of state–energy band alignment model incorporating thermal activation energies and flat-band voltages. This analytical framework proves that the composition-dependent Fermi level positioning rigorously limits and dictates complex bias-stress instabilities, offering profound insights for designing highly stable amorphous oxide semiconductor TFTs.

## 1. Introduction

Amorphous oxide semiconductors (AOSs) such as indium zinc oxide (IZO) and indium gallium zinc oxide (IGZO) have been extensively investigated as core active layer materials for next-generation large-area displays and flexible electronics [1,2,3,4,5,6]. Their remarkable electrical properties such as high mobility, optical transparency, and excellent spatial uniformity are derived from unique conduction mechanisms mediated by the overlapping percolation of spherical metal s-orbitals [7,8,9,10,11,12,13]. In recent years, solution-processed AOS thin-film transistors (TFTs) have attracted significant attention because of their ability to facilitate low-temperature and low-cost fabrication and their potential for direct printing manufacturing. Despite these advantages, the electrical instability of solution-processed AOS TFTs under continuous bias stress (BS) remains one of the major challenges limiting their practical implementation [14,15,16,17,18]. Accurately describing the operational physics of these devices, particularly the defect state distribution, band-tail states, and charge transport under electrical stress, requires comprehensive structural and electrostatic analysis [19,20].

Threshold voltage (V_th_) shifts under positive bias stress (PBS) and negative bias stress (NBS) are widely employed as indicators of device degradation [21,22,23]. This pronounced threshold voltage shift is a widely recognized degradation mechanism in amorphous oxide semiconductors, frequently observed in macroscopic BS evaluations [24,25]. While many stability analyses frequently relied on macroscopic time-dependent V_th_ tracking, numerous investigations have successfully combined standard electrical characterizations with advanced spectroscopic and defect analyses to elucidate these degradation pathways. A significant aspect of these mechanistic studies involves the complex thermodynamic and charge-exchange characteristics of oxygen vacancies (V_O_) within the oxide matrix [26,27,28,29,30]. As demonstrated in foundational effect studies [31,32,33], oxygen vacancies can exist in three distinct charge states (V_O_^0^, V_O_^+^, and V_O_^2+^). The associated physical mechanisms involve dynamic charge exchange with both electrons from the localized defect states, resulting in highly conditional ionization and trapping processes depending on the applied electrical field and external energy, providing a critical theoretical basis for evaluating instability degradation.

Although these studies have significantly improved the understanding of bias-stress degradation, studies have predominantly focused on individual degradation mechanisms or correlating device instability with static electrical characteristics. Recent DOS-based investigations have further provided valuable insights regarding the distribution of electronic gap states and their influence on carrier transport [34,35,36,37]. Nevertheless, the relationship between DOS distributions and the dynamic evaluation of reversible and irreversible degradation processes under BS remains insufficiently understood. Consequently, an analytical framework capable of linking experimentally extracted DOS distributions with time-dependent electrical characteristics remains highly necessary.

While previous DOS-based investigations have predominantly focused on characterizing electrostatic charge transitions or employing gap-state distributions to describe generalized trapping mechanisms, the novelty of this study lies in integrating an experimentally extracted DOS framework with an interfacial energy band alignment model to analyze the dynamics of BS degradation. The key parameters defining the energy band alignment, including the indium molarity-dependent structural characteristics, Fermi level positions, and the independently extracted acceptor-like and donor-like state distribution obtained through thermal- and photon-energy-based analyses, were experimentally established in our previous studies [38,39]. Rather than interpreting TFT instability solely through macroscopic threshold voltage shifts, we systematically decouple reversible interactions correlated with the leakage current and irreversible bulk trapping in solution-processed IZO TFTs by tracking defect kinetics under BS conditions. In this study, fundamental electrical stabilities under zero-BS, PBS, and NBS conditions were comprehensively evaluated as functions of the indium molarity ratio. Extracting the time-dependent transfer curves and saturation leakage current variations revealed a highly decoupled behavior, suggesting that PBS is predominantly governed by a reversible neutralization process that screens the effective carriers, whereas NBS originates from irreversible bulk trapping via the ionization of V_O_. Finally, we present a complete DOS–energy band alignment model that can describe BS instabilities through charge transitions between gap-state distributions (InO-related shallow tail states and ZnO-related V_O_). This analytical framework quantitatively clarifies how compositional control dominates the transition from reversible to irreversible degradation and provides a theoretical guideline for improving the electrical reliability of solution-processed oxide TFTs.

## 2. Materials and Methods

### 2.1. Preparation of IZO Precursor Solutions

IZO precursor solutions were prepared by dissolving metal nitrates in 2-methoxyethanol (2-ME, Sigma-Aldrich, St. Louis, MO, USA). The zinc nitrate hydrate (Zn(NO_3_)_2_∙xH_2_O, Sigma-Aldrich, St. Louis, MO, USA) precursor was dissolved to maintain a fixed molarity of 0.25 M. The molarity of the indium precursor (In(NO_3_)_3_∙xH_2_O, Sigma-Aldrich, Burlington, MA, USA) was varied to 0.0125, 0.025, 0.05, 0.1, 0.125, 0.15, and 0.2 M to systematically investigate the effect of the indium concentration on the DOS and BS instability. The prepared solutions were stirred using a magnetic bar at 60 °C for over 6 h to ensure complete ionization and homogeneously mix sol states prior to thin-film deposition.

### 2.2. Device Fabrication via the Sol–Gel Process

**Figure 1:**
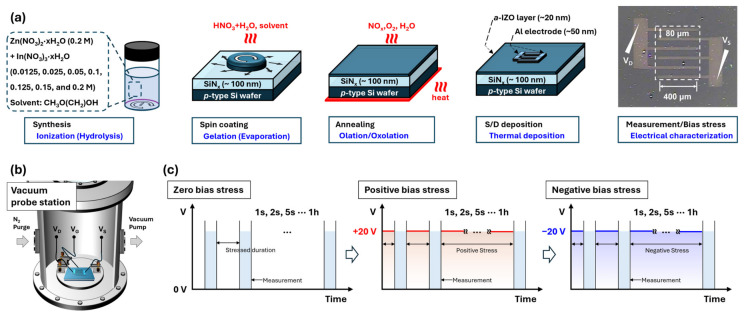
Schematic of the fabrication procedure and electrical characterization methodology for solution-processed IZO TFTs. (**a**) Sol–gel synthesis and device integration steps including spin coating, thermal annealing, and electrode deposition. (**b**) The experimental setup of the vacuum probe station for BS evaluation. (**c**) Sequential measurement step of the BS protocol under zero-BS, PBS and NBS conditions.

Bottom-gate top-contact IZO TFTs were fabricated on a *p*-type silicon (*p*-Si) wafer onto which a 100 nm thick silicon nitride (SiN_x_) layer was sputtered to serve as the gate dielectric. As indicated in Figure 1a, synthesized IZO precursor solutions are spin-coated onto O_2_-plasma-treated *p*-Si/SiN_x_ substrates. Subsequently, the spin-coated films were subjected to a pre-baking process for solvent evaporation (gelation), followed by a high-temperature thermal annealing step at 650 °C for facilitating olation/oxolation and forming a dense amorphous IZO semiconductor layer. Finally, aluminum source and drain electrodes were thermally deposited through a metal shadow mask under a base pressure of ~10^−6^ Torr. Based on the optic device image, the fabricated TFTs featured a five-finger channel structure, and the effective channel width (W) and length (L) were defined as 400 and 80 µm, respectively.

### 2.3. Electrical Characterization and BS Sequence

All electrical characterizations and BS tests were performed in a dark vacuum probe station using a semiconductor parameter analyzer (Keithley 4200A-SCS, Tektronix, Beaverton, OR, USA) (Figure 1b) to prevent electrical degradation and hysteresis induced by ambient air and moisture molecules. The time-dependent electrical stabilities of the IZO TFTs were evaluated using a precise BS protocol (Figure 1c). The initial transfer characteristics were measured in pristine states (zero BS). Subsequently, PBS and NBS were applied to the gate electrode at constant voltages of V_G_ = +20 V and V_G_ = −20 V, respectively. The zero BS and PBS characterizations were intentionally evaluated prior to the NBS sequence because NBS induces predominantly irreversible consequences in devices with a high In concentration. The transfer curves were measured between stress phases to systematically track the reversible and irreversible degradation kinetics.

## 3. Discussion

### 3.1. Composition-Dependent BS Instabilities

**Figure 2:**
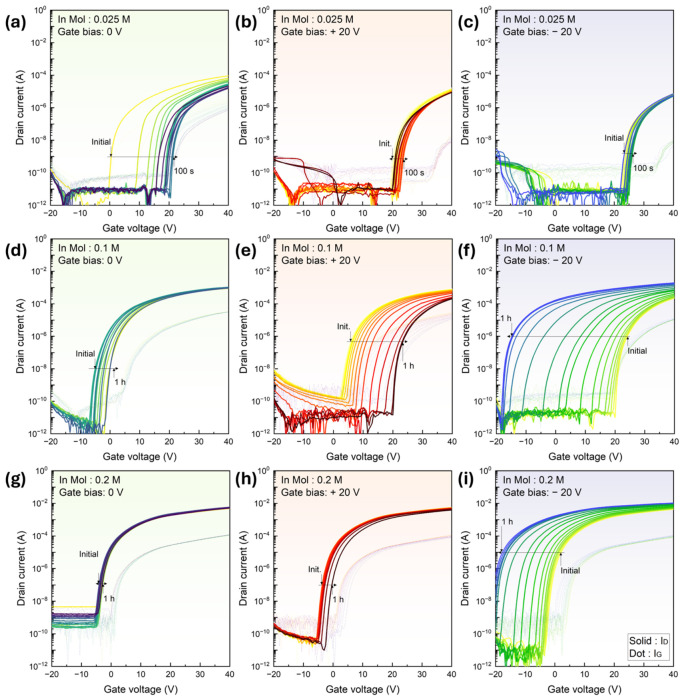
Bias-stress-induced variations in the transfer curves of solution-processed IZO TFTs under zero BS (V_G_ = 0 V), PBS (V_G_ = 20 V), and NBS (V_G_ = −20 V) conditions. Time-dependent shifts are evaluated for devices with varying indium concentrations formulated with a fixed 0.25 M zinc precursor: (**a**–**c**) low (0.025 M), (**d**–**f**) moderate (0.1 M), and (**g**–**i**) high (0.2 M). The color gradients represent the temporal evolution of the transfer curves from the initial to the final measurement, with the arrow indicating the direction of change. The solid and dotted lines represent the drain current (I_D_) and gate leakage current (I_G_), respectively, measured at a constant drain voltage of V_D_ = 20 V.

Figure 2 shows the time-dependent transfer characteristics (I_D_ − V_G_) of the solution-processed IZO TFTs subjected to zero BS (V_G_ = 0 V), PBS (V_G_ = +20 V), and NBS (V_G_ = −20 V) for 1 h. The IZO TFTs were categorized analytically according to their indium precursor molarities, which are low (0.025 M), moderate (0.1 M), and high (0.2 M). Detailed structural inspections of the deposited layers and electrical characteristics as a function of the indium molarity ratio have been comprehensively investigated and can be confirmed in our previous work [38]. These results indicate that the degradation kinetics and direction of the threshold voltage shift (ΔV_th_) are strongly dependent on the indium concentration and polarity of the applied BS. For the detailed specifications of the time-dependent degradation across the full range of indium molarities (0.0125 M to 0.2 M), the corresponding transfer curves under zero BS, PBS and NBS are presented in Figure S1.

Although the overall current levels of the low-indium-doped TFTs (Figure 2a–c) remain stable under PBS and NBS, the devices exhibit a distinct charge-neutralizing effect during initial measurement under zero-BS conditions (Figure 2a). The initial minor shift is rapidly recovered through the detrapping of charges via a reversible relaxation process. The IZO TFTs exhibit severe electrical instability across all BS conditions when the indium concentration increases to a moderate level (0.1 M) (Figure 2d–f). The TFT is notably affected by the PBS (Figure 2e), exhibiting a pronounced positive V_th_ shift. This confirms that TFTs with moderate doping can be shifted in both positive and negative directions by PBS and NBS, respectively.

Conversely, in the highly doped IZO TFTs (0.2 M, Figure 2g–i), the PBS-induced positive shift is significantly suppressed because of the predominant irreversible trapping at heavily doped indium concentrations. Further, these high-indium TFTs become more susceptible to NBS (Figure 2i) and exhibit a pronounced negative V_th_ shift. This negative shift is a widely recognized degradation mechanism in amorphous oxide semiconductors and originates primarily from the ionization of V_O_.

A crucial observation in these transient characteristics lies in the correlation between the subthreshold drain and leakage currents. Under zero-BS and PBS conditions, the onset voltage of leakage current rigidly shifts in correlation with the drain current turn-on voltage. This synchronized parallel shift confirms that the positive ΔV_th_ is dominated by electrostatic screening attributed to charge transition at the dielectric or SiN_x_/IZO interface. In contrast, the subthreshold drain current shifts negatively under NBS conditions, especially in moderately and highly doped devices (Figure 2f,i); however, the turn-on voltage of the leakage current does not synchronously follow this transition. This decoupled behavior based on the indium concentration strongly substantiates that degradations under the zero-BS, PBS, and NBS degradation are not governed by a simple electrostatic mechanism; instead, they are governed by charge transitions involving structural and energy state modifications that independently alter channel conductivity.

### 3.2. Quantitative ΔV_th_ Analysis

These current-modulation characteristics are present in the summarized electrical parameters of the IZO TFTs in terms of indium molarity. The time-dependent square root of the drain current versus gate voltage ($\sqrt{I_{D}}$ − V_G_) curves were employed to precisely extract the threshold voltage variations, as presented in Figure S2. Moreover, detailed time-dependent ΔV_th_ profiles across the entire indium molarity range (0.0125–0.2 M) under all BS conditions are provided systematically in Figure S3.

**Figure 3:**
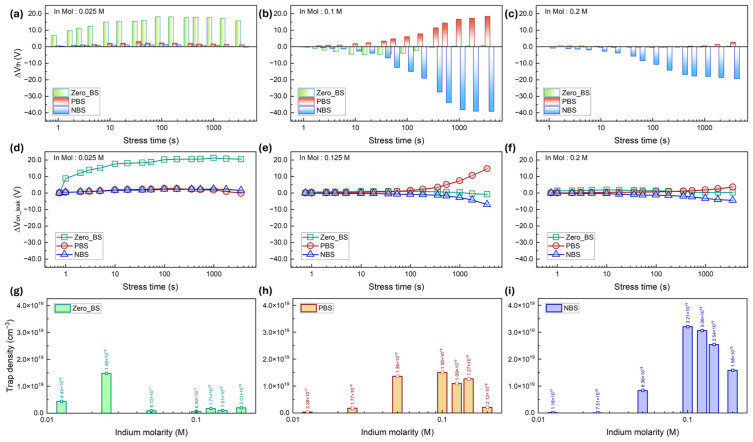
Quantitative analysis of BS instabilities and corresponding trap states in IZO TFTs. Time-dependent shifts in (**a**–**c**) the threshold voltage ΔV_th_, and (**d**–**f**) the onset voltage of leakage current ΔV_on_leak_ for devices with low (0.025 M), moderate (0.1 M), and high (0.2 M) indium concentrations. (**g**–**i**) Extracted effective trap densities ΔN_Trap_ derived from the ΔV_th_ variations plotted as a function of the indium molarity ratio (0.0125 M to 0.2 M) under the respective zero-BS, PBS, and NBS conditions.

Figure 3a–f show the extracted ΔV_th_ and turn-on voltage shifts in leakage current (ΔV_on_leak_) for the representative low (0.025 M)-, moderate (0.1 M)-, and high (0.2 M)-indium-doped TFTs. Detailed time-dependent ΔV_on_leak_ characteristics based on indium molarity can be confirmed in Figure S4. As observed in the transfer curves, the matching parallel shifts in ΔV_th_ and ΔV_on_leak_ under PBS validate a purely electrostatic charge-transition mechanism via the dielectric interface. In contrast, the highly decoupled behavior between ΔV_th_ and ΔV_on_leak_ under NBS indicates that degradation is governed by a bulk-dominated structural transition instead of a dielectric interface that induces screening. Moreover, the time-dependent saturation leakage current (V_D_ = +40 V, V_G_ = +40 V) presented in Figure S5 reveals an asymmetric disproportionate response in high-In TFTs. Although the ΔV_th_ shift induced by NBS is more than twice as large as that by PBS, the resulting variation in the saturation leakage current under NBS remains comparable to or smaller than the modulation observed under PBS.

As summarized in Table 1, effective areal trap densities (ΔN_ts_, [cm^−2^]) are extracted from ΔV_th_ to quantitatively evaluate inherent kinetics and convert them into the corresponding volumetric total trap densities (ΔN_t_, [cm^−3^]) depicted in Figure 3g–i. The areal trap density was calculated using the fundamental relationship

(1) $$V_{th}=V_{FB}+2\phi_{F}+\frac{\sqrt{2q\epsilon_{o}\epsilon_{s}N_{A}\left(2\phi_{F}\right)}}{C_{ox}}-\frac{Q_{t}}{C_{ox}},\;\Delta V_{th}=-\frac{\Delta Q_{t}}{C_{ox}}=-\frac{q\cdot \Delta N_{ts}}{C_{ox}},$$

where C_ox_ and q represent the capacitance per unit area of the SiN_x_ gate dielectric and electric charge, respectively. In this calculation, an effective channel accumulation thickness of 5 nm is defined based on the assumption of a charge-sheet approximation. Under NBS conditions, degradation is an inherent bulk process corresponding to the depletion region, which extends across the entire ~20 nm thickness of the semiconductor film. Conversely, under PBS conditions, the threshold voltage shift is predominantly driven by interfacial trapping confined within the Debye screening length. Because the threshold voltages are extracted via the linear extrapolation of the $\sqrt{I_{D}}$ − V_G_ curve in the saturation regime, we have assumed that the associated trapping charges are uniformly distributed within this relatively thin 5 nm normalized layer. Additionally, the areal charge density due to the V_th_ shift is presented in Table S1.

The magnitude of the extracted total trap density ΔN_t_ quantitatively proves the proposed degradation mechanisms. Under the zero-BS condition, a maximum volumetric trap density of 1.48 × 10^19^ cm^−3^ was observed in IZO TFTs with relatively low indium concentrations. Charge neutralization rearrangement during the initial measurements of low-indium-doped IZO TFTs suggests that the semiconductor is negatively polarized under long-term storage conditions. A comparable amount (~1.36 × 10^19^ cm^−3^) of total trap density is observed under the PBS condition in the moderate-indium-doping region. This effect does not appear in low-In TFTs and indicates that effective negative charges correlated with In react with PBS. Conversely, under the NBS condition, N_t_ increases dramatically in the indium-rich devices, peaking at 3.21 × 10^19^ cm^−3^. This massive and monotonic generation of effective negative charges is consistent with the extensive ionization of electrons from V_O_ within the indium-rich bulk IZO matrix.

The total density of gap states is evaluated by integrating the exponentially distributed acceptor-like states to fundamentally comprehend the origin of BS instabilities. This approach indicates that the extracted DOS distribution is a highly efficient analytical tool for understanding TFT instability. Based on our previous study [39], the overall gap-state distribution within the amorphous IZO semiconductor can be structurally decoupled: deep acceptor-like states can be correlated with the ZnO amorphous random network while the shallow acceptor-like states (band-tail states) originated from the crystalline InO sub-lattice. The density of the exponentially distributed acceptor-like states (N_ta_(E), [cm^−3^∙eV^−1^]) can be modeled using

(2) $$N_{ta}\left(E\right)=N_{ta\_deep}\exp\left(-\frac{E_{C}-E}{kT_{c\_deep}}\right)+N_{ta\_shallow}\exp\left(-\frac{E_{C}-E}{kT_{c\_shallow}}\right),$$

where N_ta_ and kT_c_ represent the density of acceptor-like trap states at the conduction band edge and the characteristic energy (distribution slope) for the deep and shallow states, respectively. In addition, donor-like states located below the Fermi level (E_F_) are intrinsically associated with ZnO V_O_. A Gaussian distribution function is employed to model these states broadly. The density of oxygen vacancy-related states N_Vo_(E) can be mathematically described as

(3) $$N_{V_{O}}\left(E\right)=N_{V_{O}}\exp\left[-{\left(\frac{E-E_{V_{O}}}{\sigma_{V_{O}}}\right)}^{2}\right],$$

where N_Vo_, E_Vo_, and σ_Vo_ represent the maximum peak density of oxygen vacancy states, central peak energy position of the distribution, and characteristic constant defining the standard deviation (energy broadness) of the Gaussian profile, respectively. The specific parameters of these gap states employed via simple charge-sheet and field-effect analysis methods [9,39] are summarized in Table S2.

The theoretical total amount of available gap states (N_t_deep_, N_t_shallow_) was calculated by integrating the acceptor-like trap distribution from E_F_ to E_C_ to quantitatively correlate this continuous energy distribution with the total capacity for electron trapping under BS. The integration is expressed as

(4) $$N_{total}=\int_{E_{F}}^{E_{C}}N_{ta}\left(E\right)dE.$$

The total integrated trap densities are obtained by calculating this integration for the deep- and shallow-state components, as presented in Table 2.

A direct quantitative correlation is observed between these integrated DOS capacities (Nt_otal_) and experimentally extracted effective trap densities (ΔN_t_) derived from the charge densities and threshold voltage shifts (Table 1, Figure 3g–i). For the low-doped IZO TFTs under the zero-BS condition (Figure 3g), the extracted trap density was relatively balanced with the calculated total number of deep acceptor-like states. The density of the shallow-band-tail states increased significantly with an increase in the In molarity ratio. Consequently, the extracted ΔN_t_ correlates with the integrated total density of shallow states N_t_shallow_ for the moderate-indium TFTs under PBS (Figure 3h), which proves that PBS-induced electrons are predominantly collapsed by InO-related tail states.

A profound insight into the DOS distribution emerged under NBS conditions in highly doped TFTs [40,41]. Although the NBS-induced degradation is initiated by the ionization of donor-like V_O_ (V_O_ → V_O_^+^ + e^−^), the resulting transition modulates the drain current through electrical interactions with acceptor-like states near E_C_. The number of InO-related tail states increased continuously with increasing indium concentration, whereas the number of ZnO-related V_O_ fundamentally decreased because of the replacement proportion of the ZnO network. Despite possessing the largest number of shallow states acting as potential electron reservoirs, the actual total shift caused by ΔN_t_ decreases at the highest indium concentration of 0.2 M (Figure 3i). This confirms that the extent of NBS degradation is limited by the number of ZnO V_O_, suggesting that the DOS distribution profile accurately dictates the composition-dependent electrical instability of IZO TFTs.

**Figure 4:**
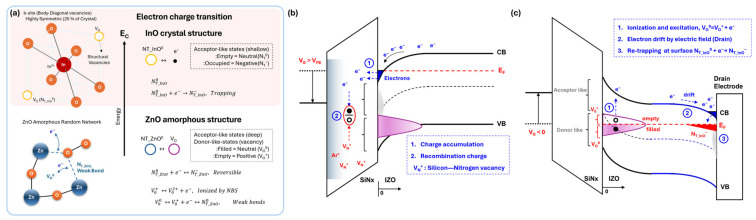
Comprehensive physical mechanisms of bias-stress-induced instabilities in IZO TFTs. (**a**) Schematic of idealized local atomic structures and charge-transition mechanisms for the crystalline InO and amorphous ZnO sub-lattices. Energy band diagrams of the SiN_x_/IZO interface illustrating the band bending and degradation kinetics under (**b**) PBS and (**c**) NBS conditions.

The atomic structure model and corresponding energy band diagrams at the SiN_x_/IZO interface are shown in Figure 4 to comprehensively understand the defect kinetics governing BS instabilities. Figure 4a presents the idealized local atomic structures of the crystalline InO and amorphous ZnO sub-lattices along with their electron charge-transition mechanisms based on their respective energy levels [42,43,44]. For clarity in structural mapping, the previously defined shallow acceptor-like (N_ta_shallow_), deep acceptor-like (N_ta_deep_), and oxygen vacancy states (N_Vo_) are denoted as N_T_InO_, N_T_ZnO_, and V_O_, respectively. In typical unipolar metal–oxide semiconductors such as IZO, transport is exclusively dependent on electrons because the minority carrier (hole) mobility is negligible when elaborating on drain current modulation. Illustrated electron transition mechanisms accurately reflect the acceptor-like trapping nature of N_T_InO_ and N_T_ZnO_ (N_T_^0^ + e^−^ → N_T_^−^) and donor-like ionization behavior of V_O_ (V_O_^0^ → V_O_^+^ + e^−^). Based on practical observations derived from the threshold voltage shift results, ZnO-related deep traps (N_T_ZnO_) and V_O_ exhibit an exchangeable and reversible charge-state relationship (N_T_ZnO_^−^ ↔ V_O_^+^ + e^−^), and this can be attributed to the adjustable amorphous random network of the ZnO structure. In contrast, the nearest band-tail states (N_T_InO_) intrinsically linked to the highly coordinated InO b- and d-site vacancies exhibit irreversible electron trapping because of their strong crystalline bonding nature.

Figure 4b shows a schematic of the PBS effect by depicting energy band bending across the SiN_x_/IZO interface. Under a positive gate bias, downward band bending induces a substantial accumulation of electrons in the channel. We speculate that the reduction in effective negative charges in the semiconductor triggers the positive ΔV_th_, which originates from a leakage-current-assisted recombination process at the dielectric interface. The accumulated electrons at the interface recombine with pre-existing positive impurities within the SiN_x_ dielectric, which leads to the neutralization of acceptor-like trap states and a consequential positive V_th_ shift. The leakage current via this recombination process (R_R_) can be mathematically expressed as

(5) $$R_{R}=-\sigma_{n}\nu_{th}N_{t}\Delta n\equiv -\frac{\Delta n}{\tau_{R}},$$

where σ_n_, ν_th_, N_t_, Δn, and τ_R_ represent the electron capture cross-section, thermal velocity, density of traps, excess electron concentration, and recombination lifetime, respectively. The continuous reduction in the measurement leakage current directly signifies a reduction in the available trap density (N_t_) because the accumulated excess carrier density (Δn) remains constant under a fixed gate/drain bias. Positive impurities in the dielectric such as nitrogen vacancies (V_N_^+^) and argon ions (Ar^+^) are inevitably introduced by structural defects in the SiN_x_ matrix and in the Ar plasma during the sputtering deposition process.

Conversely, Figure 4c shows degradation kinetics under NBS conditions, wherein the negative gate bias bends the energy band of the semiconductor upward. The degradation proceeds through a three-step sequential mechanism: (1) Donor-like oxygen vacancy states are ionized (V_O_^0^ → V_O_^+^ + e^−^), which generates excess free electrons. (2) The generated electrons are swept toward the surface and drift laterally toward the drain electrode because of the applied electric field. (3) A significant portion of these electrons is subsequently re-trapped at the semiconductor surface, increasing the shallow tail state level near the E_C_ and inducing a heavily accumulated (metal-like) channel state that manifests as severe negative charges at the interface and an irreversible V_th_ shift.

To further support that this NBS degradation is primarily governed by oxygen vacancy ionization rather than interfacial charge injection, this mechanism needs to be correlated with the irreversible trapping dynamics of the InO sub-lattice. As independently demonstrated through recent retention characterization utilizing thermal and photo post-treatments [39], the extensive irreversible behavior of the NBS-induced shift in highly indium-doped TFTs is directly attributed to the subsequent trapping of these ionized electrons within the crystalline InO-related shallow tail states.

### 3.3. DOS–Energy Band Alignment

**Figure 5:**
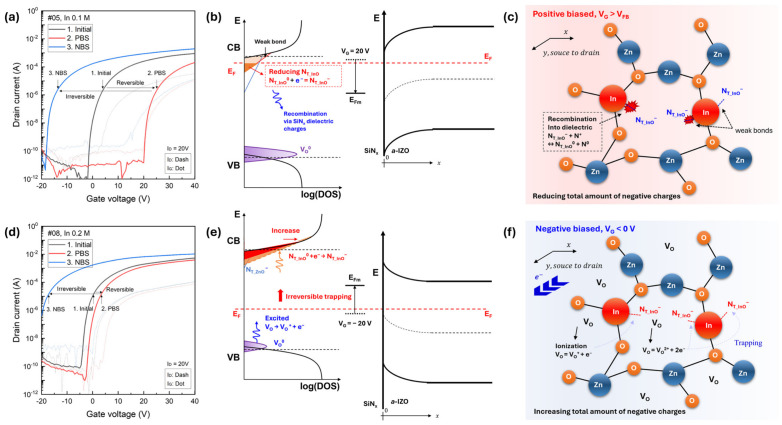
Reversible and irreversible degradation kinetics in the moderate- and high-indium-doped IZO TFTs. (**a**) Transfer characteristics of the moderate-doped TFT exhibiting fluctuating threshold voltage shifts under alternating PBS and NBS conditions. (**b**) Corresponding energy band diagram and DOS distribution at the SiN_x_/IZO interface under PBS, which illustrates band bending. (**c**) Schematic of the recombination process that reduces effective negative charges at InO trapping sites. (**d**–**f**) Corresponding analysis for high-doped IZO TFT depicting the severe negative shift and underlying irreversible electron trapping mechanisms caused by ionized ZnO V_O_.

Although detailed energy band diagrams across dielectric and IZO semiconductors provide a theoretical understanding of underlying mechanisms, DOS profiles are significantly more effective for explaining the origins of instabilities arising from the bulk semiconductor and interface. From this perspective, the DOS–energy band alignment is a highly useful method to verify bulk instability, which includes interfacial effects. Figure 5 demonstrates how this DOS–energy band diagram alignment effectively interprets the instability of TFT devices. The V_th_ instability is defined by transfer characteristics to construct this analytical model, as indicated in Figure 5a. Subsequently, energy band bending under a specific BS (e.g., PBS in Figure 5b) is depicted, and the DOS profile is positioned at the E_C_ and E_V_ edges. The Fermi level (E_F_) is set based on the flat-band conditions of the bulk semiconductor. Specific activation energies used for estimating E_F_ levels according to the indium molar ratio are shown in Figure S6. These thermal activation energies are extracted based on the thermal analysis of a simple charge approximation and field-effect analysis [31]. The reversible and irreversible transitions within the DOS profile can be clearly defined by tracking charge transitions based on the fact that electrons occupy states below the E_F_ level.

The device exhibited both InO and ZnO characteristics when applying this methodology to the moderately indium-doped TFT (Figure 5a–c), which reacted as a counter effect under BS. As shown in Figure 5a, the TFT exhibits fluctuating ΔV_th_ shifts under alternating PBS and NBS conditions. Figure 5b illustrates downward band bending in PBS and the corresponding DOS alignment. In this regime, the accumulated electrons fill the available acceptor-like states; however, simultaneous recombination via dielectric charges reduces the effective negative charges at InO trapping sites (Figure 5c). This neutralization acts as a countereffect, thereby leading to the reversible and fluctuating nature of the threshold voltage.

In contrast, Figure 5d–f present an interpretation of the NBS-dominated behavior in highly indium-doped TFTs. Under a negative gate bias, upward band bending excites donor-like states, initiating the ionization of V_O_. The DOS–energy band alignment depicts a large amount of irreversible electron trapping within the high-density shallow-band-tail states continuously supplied by ionized V_O_. This massive generation and subsequent trapping in the N_t_InO_ state profoundly increases the total number of negative charges at the interface. The corresponding transitions in the atomic structure are also illustrated in Figure 5f to comprehensively explain the severe and irreversible ΔV_th_ shift observed in the high-indium-doped IZO TFTs.

**Figure 6:**
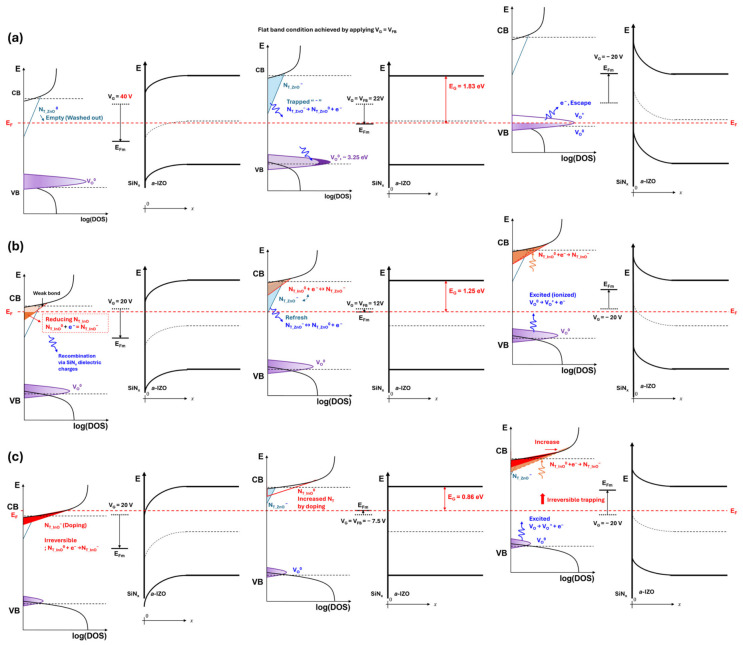
Energy band diagrams and corresponding gap-state distributions at the SiN_x_/IZO interface under PBS, zero-BS, and NBS conditions. Systematic changes in the band alignment and underlying defect kinetics are illustrated for the (**a**) low-, (**b**) moderate-, and (**c**) high-indium-doped IZO TFTs. The schematics show Fermi level positioning, electrostatic band bending, and dominant charge-transition mechanisms driving BS instabilities.

A systematic analysis using the complete DOS–energy band alignment is performed to summarize BS instability (Figure 6). In the schematics shown in Figure 6, electrostatic band bending is precisely evaluated by considering the applied BS (V_G_ = ± 20 V) along with the distinct flat-band voltages (V_FB_ of 22 V, 12 V, and −7.5 V) and thermal activation energies (E_a_ of 1.83 eV, 1.21 eV, and 0.86 eV) for the low-, moderate-, and high-indium-doped IZO TFTs, respectively. Moreover, the model explicitly incorporates the zero-BS displacement charge arrangement, which is initially polarized to compensate for pre-existing charges attributed to positive impurities within the dielectric accumulated during long-term storage conditions. The initially trapped removable charge and irreversibly trapped charge states can be depicted by the filled DOS area that corresponds to empty/occupied states in this diagram. In Figure 6, it should be noted that the proposed DOS–energy band alignment model has several inherent limitations. The key parameters, including the gap-state distributions, Fermi level (E_F_), and flat-band voltage (V_FB_) at different indium concentrations, were experimentally determined and validated in our previous studies. However, the calculated trapped charge densities do not completely coincide with the quantitatively integrated gap-state distributions because the model is intended to provide a qualitative description of charge-transition behavior. In addition, the threshold voltage shift (ΔV_th_), extracted by linear extrapolation of the $\sqrt{I_{D}}$ − V_G_ curve, is an indirect measure of localized defect states. Despite these limitations, the proposed methodology effectively visualizes charge-transition kinetics and provides a consistent interpretation of the different mechanisms governing reversible and irreversible bias-stress instability. Therefore, the notable instability features for each doping regime can be fundamentally verified by integrating E_F_ positioning with these DOS–band alignment profiles.

To systematically summarize these phenomenological observations and theoretical correlations, Table 3 provides a comprehensive explanation of the electrostatic mechanisms governing device instability across the evaluated regimes. This chart correlates the dominant degradation pathway and its physical microstructural origin with the maximum extracted trap density, validating the transition from reversible interfacial recombination to irreversible bulk trapping. In principle, precisely examining the macroscopic causes of BS instability through decoupled methodology establishes a fundamental foundation for optimizing the electrical reliability of AOS TFTs. For instance, under PBS, variations in plasma deposition or post-deposition treatment modulate the density of pre-existing positive structural defects within the dielectric, such as nitrogen vacancies (V_N_^+^) and trapped gas impurity ions (Ar^+^). As demonstrated in recent studies, targeted post-deposition treatments and dielectric engineering [19,20] can effectively optimize the dielectric stoichiometry to reduce these interfacial recombination centers. Likewise, this mechanistic insight is equally applicable to NBS-induced instability by identifying the contribution of irreversible bulk trapping associated with the crystalline InO sub-lattice. Consequently, a rigorous understanding of these decoupled charge-transition mechanisms provides an essential inspection methodology for mitigating TFT instability and enhancing overall TFT performance.

## 4. Conclusions

This study systematically decoupled the origins of BS instabilities in solution-processed IZO TFTs into reversible charge exchange at the dielectric interface and irreversible trapping in bulk shallow acceptor-like states. We analyzed the time-dependent transfer characteristics across a wide range of indium concentrations, and the results demonstrated that degradation kinetics are fundamentally affected by Fermi level positioning and localized gap-state distributions. Further, we calculated and compared ΔN_t_, N_t_deep,_ and N_t_shalow_ to validate the correlation between the BS-induced ΔV_th_ shifts and DOS profile. In PBS, instability is dominated by reversible electrostatic neutralization at the ZnO- and InO-related shallow acceptor-like states, which is dynamically counteracted by leakage current-assisted recombination between the dielectric and semiconductor. In contrast, the severe negative shift observed under NBS in highly indium-doped TFTs originated from irreversible bulk trapping triggered by the ionization of donor-like V_O_ within the amorphous ZnO sub-lattice. Moreover, the proposed DOS–energy band alignment methodology provides a direct quantitative correlation between the theoretical capacity of the gap state and experimentally extracted trap densities. The comprehensive electrostatic model not only clarifies the complex composition-dependent degradation behavior in IZO TFTs but also establishes a fundamental framework for designing highly reliable amorphous oxide semiconductor devices.

## Figures and Tables

**Table 1:** Areal effective trap densities ΔN_t_ extracted from the threshold voltage shifts in the IZO TFTs under zero-BS, PBS, and NBS conditions as a function of indium molarity ratio.

Indium Molarity (M)		0.0125	0.025	0.05	0.1	0.125	0.5	0.2
Bias stress	ΔN_ts_ZeroBS_ (cm^−2^)	2.20 × 10^12^	7.40 × 10^12^	4.36 × 10^11^	3.26 × 10^11^	8.56 × 10^11^	5.04 × 10^11^	1.00 × 10^11^
	ΔN_ts_PBS_ (cm^−2^)	1.69 × 10^11^	8.86 × 10^11^	6.80 × 10^12^	7.52 × 10^12^	5.46 × 10^12^	6.34 × 10^12^	1.06 × 10^12^
	ΔN_ts_NBS_ (cm^−2^)	5.82 × 10^10^	3.76 × 10^10^	4.18 × 10^12^	1.60 × 10^13^	1.53 × 10^13^	1.27 × 10^13^	7.92 × 10^12^

**Table 2:** Total integrated trap densities N_total_ of deep and tail states derived from the exponential distribution models in ref. [31] (simple charge sheet vs. field-effect analysis) as a function of the indium molarity ratio.

Indium Molarity (M)		0.0125	0.025	0.05	0.1	0.125	0.5	0.2
Simple charge sheet	N_t_deep_ (cm^−3^)	1.00 × 10^19^	3.30 × 10^18^	6.95 × 10^18^	6.95 × 10^18^	9.00 × 10^17^	3.09 × 10^19^	3.46 × 10^18^
	N_t_tail_ (cm^−3^)	N/A	2.92 × 10^19^	1.98 × 10^19^	2.97 × 10^19^	8.99 × 10^19^	1.41 × 10^20^	5.37 × 10^19^
Field-effect analysis	N_t_deep_ (cm^−3^)	1.31 × 10^19^	5.15 × 10^19^	1.01 × 10^20^	5.92 × 10^20^	5.35 × 10^21^	3.60 × 10^21^	3.95 × 10^21^
	N_t_tail_ (cm^−3^)	4.77 × 10^19^	1.46 × 10^20^	3.10 × 10^20^	2.06 × 10^21^	2.05 × 10^21^	2.85 × 10^22^	1.23 × 10^22^

**Table 3:** Summary of the dominant degradation mechanisms, proposed instability origins, and maximum extracted areal trap densities (ΔN_ts_) as a function of the indium molarity regime in solution-processed IZO TFTs.

Indium Molarity Regime	Dominant BS Response	Dominant Degradation Mechanism	Max Extracted Areal Trap Density (ΔN_ts_)
Low (≤0.05 M)	Zero BS (Initial State)	Displacement charge arrangement to compensate for pre-existing positive fixed impurities (V_N_^+^) within SiN_x_ dielectric	~7.40 × 10^12^ cm^−2^ (at 0.025 M under zero BS)
Moderate (~0.1 M)	PBS and NBS (Fluctuating)	Leakage-current-assisted recombination at the dielectric interface dynamically counteracted by moderated V_O_ ionization	~7.52 × 10^12^ cm^−2^ (at 0.1 M under PBS)
High (≥0.15 M)	NBS	Massive ionization of donor-like oxygen vacancies (V_O_^0^ → V_O_^+^ + e^−^) in the amorphous ZnO sub-lattice; irreversible electron trapping in crystalline InO-related shallow tail states	~1.27 × 10^13^ cm^−2^ (at 0.15 M under NBS)

## Data Availability

The original contributions presented in the study are included in the article and Supplementary Materials. Further inquiries and raw data supporting the reported results can be directed to the corresponding author upon reasonable request.

## Supplementary Materials

The following supporting information can be downloaded at https://www.mdpi.com/article/10.3390/nano16140877/s1: Table S1: Total areal charge density extracted from threshold voltage shift; Table S2. Acceptor-like states distribution in terms of the doping concentration, Ref. [39]; Figure S1: Time-dependent transfer characteristics of the solution-processed IZO TFTs; Figure S2: Time-dependent square root of drain current versus gate voltage ($\sqrt{I_{D}}$ − V_G_); Figure S3: Threshold voltage shifts versus time; Figure S4: Turn-on voltage shift in the leakage current (ΔV_on_leak_) versus graph of solution-processed IZO TFTs; Figure S5: Leakage current variations versus time graphs of the solution-processed IZO TFTs; Figure S6: Energy band alignments and Fermi level positioning.

## Abbreviations

The following abbreviations are used in this manuscript:

AOSs	Amorphous oxide semiconductors
IZO	Indium zinc oxide
IGZO	Indium gallium zinc oxide
TFT	Thin-film transistor
PBS	Positive bias stress
NBS	Negative bias stress
BS	Bias stress
V_o_	Oxygen vacancies
DOS	Density of states
