# Supplementary material for: Decoupling Reversible Interface Trapping and Irreversible Bulk Transitions in Solution-Processed Indium Zinc Oxide Thin-Film Transistors
Source: Nanomaterials (Basel). 2026 Jul 16;16(14):877. doi: 10.3390/nano16140877 (PMC13415042; doi:10.3390/nano16140877)
Supplement: Supplementary file 1 [file nanomaterials-16-00877-s001.zip › nanomaterials-4417022-supplementary.pdf]

Supplementary Materials

Tables

Table S1. Total areal charge density extracted from threshold voltage shift

Table S2. Acceptor-like states distribution in terms of the doping concentration. Ref. [39]

Figures

Figure S1. Time-dependent transfer characteristics of solution-processed IZO TFTs under (a) PBS (V_G = +20 V, top row), (b) zero BS (V_G= 0V, middle row), and (c) NBS (V_G = − 20 V, bottom row) conditions. Systematic variations in transfer curves are plotted as a function of the indium molarity ratio, increasing from 0.0125 M to 0.2 M (from left to right)

Figure S2. Time-dependent square root of drain current versus gate voltage ($\sqrt{I_D}$–V_G) curves of solution-processed IZO TFTs under (a) PBS, (b) zero BS, and (c) NBS conditions. The curves are plotted as a function of the indium molarity ratio, increasing from 0.0125 M to 0.2 M (from left to right)

Figure S3. Threshold voltage shifts versus time graph of the solution-processed IZO TFT under bias zero BS, PBS, and NBS conditions. Extracted ΔV_{th} variations are systematically presented for devices varying indium molarity ratio in (a–g), increasing from 0.0125 M to 0.2 M, respectively.

Figure S4. Turn-on voltage shift of the leakage current (ΔV_{on_leak}) versus graph of solution-processed IZO TFTs under zero BS, PBS, and NBS conditions. Extracted ΔV_{on_leak} variations are systematically presented for devices with varying indium molarity ratios in (a–g), increasing from 0.0125 M to 0.2 M, respectively.

Figure S5. Leakage current variations versus the time graphs of solution-processed IZO TFTs measured under saturation conditions (V_D = +40 V, V_G = +40 V). Extracted leakage currents under zero BS, PBS, and NBS conditions are systematically presented for devices based on the indium molarity ratios in (a–g), increasing from 0.0125 M to 0.2 M, respectively.

Figure S6. Energy band alignments and Fermi level positioning derived from thermal activation energy extractions. (a) Schematic of the temperature-dependent measurement setup used to extract activation energy. (b) Energy band diagram defining the fundamental principle of thermal activation energy E_a in relation to Fermi level E_F. (c) Systematic changes in energy band alignments demonstrating the rise of $E_F - E_i$ toward E_C as a function of increasing indium concentration (from sample #02 to #08).

Table S1. Total areal charge density extracted from threshold voltage shift								
Indium Molarity (M)		0.0125	0.025	0.05	0.1	0.125	0.15	0.2
Bias Stress	Q_{t_ZeroBS} ($C \cdot cm^{-2}$)	8.54×10^{-6}	2.96×10^{-5}	-5.91×10^{-6}	-1.30×10^{-6}	-3.43×10^{-6}	2.02×10^{-6}	4.02×10^{-6}
	Q_{t_PBS} ($C \cdot cm^{-2}$)	-6.77×10^{-7}	3.54×10^{-6}	2.73×10^{-5}	3.01×10^{-5}	2.19×10^{-5}	2.54×10^{-5}	4.25×10^{-6}
	Q_{t_NBS} ($C \cdot cm^{-2}$)	2.33×10^{-7}	1.50×10^{-7}	-1.67×10^{-5}	-6.42×10^{-5}	-6.13×10^{-5}	-5.09×10^{-5}	-3.17×10^{-5}

(Table S1.)

Table S2. Acceptor-like states distribution in terms of the doping concentration. Ref. [39]								
Indium molarity (M)		0.0125	0.025	0.05	0.1	0.125	0.5	0.2
Activation energy, E_a (eV)		1.83	1.55	1.49	1.21	1.25	1.06	0.86
Simple charge sheet	$N_{ta\_deep}(E)$ (eV^{-1} cm^{-3})	1.83×10^{19}	5.26×10^{18}	1.50×10^{19}	1.63×10^{19}	1.61×10^{18}	7.99×10^{19}	9.83×10^{18}
	kT_{c_deep} (meV)	572	707	486	459	657	421	397
	$N_{ta\_shallow}(E)$ (eV^{-1} cm^{-3})	N/A	1.77×10^{20}	8.13×10^{19}	2.12×10^{20}	2.81×10^{21}	3.27×10^{21}	1.22×10^{21}
	$kT_{c\_shallow}$ (meV)	N/A	165	244	140	32	43	44
Field-effect analysis	$N_{ta\_deep}(E)$ (eV^{-1} cm^{-3})	1.77×10^{19}	1.17×10^{20}	1.99×10^{20}	1.75×10^{21}	1.66×10^{22}	1.49×10^{22}	2.08×10^{22}
	kT_{c_deep} (meV)	829	455	539	349	330	245	192
	$N_{ta\_shallow}(E)$ (eV^{-1} cm^{-3})	1.70×10^{20}	1.13×10^{21}	1.65×10^{21}	2.42×10^{22}	1.95×10^{22}	4.45×10^{23}	4.23×10^{23}
	$kT_{c\_shallow}$ (meV)	281	129	188	85	105	64	29

(Table S2.)

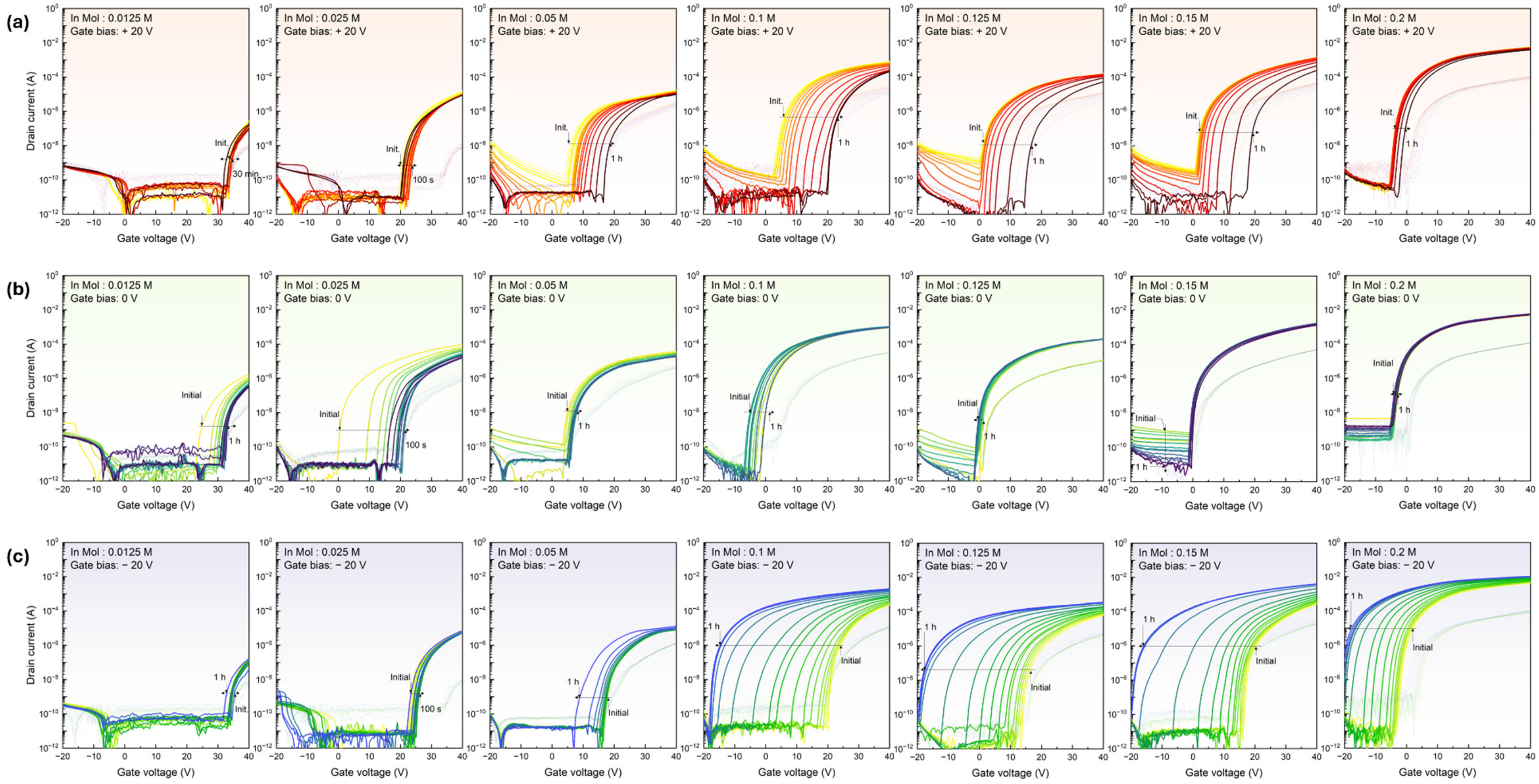

(a)
In Mol : 0.0125 M
Gate bias: + 20 V
In Mol : 0.025 M
Gate bias: + 20 V
In Mol : 0.05 M
Gate bias: + 20 V
In Mol : 0.1 M
Gate bias: + 20 V
In Mol : 0.125 M
Gate bias: + 20 V
In Mol : 0.15 M
Gate bias: + 20 V
In Mol : 0.2 M
Gate bias: + 20 V
(b)
In Mol : 0.0125 M
Gate bias: 0 V
In Mol : 0.025 M
Gate bias: 0 V
In Mol : 0.05 M
Gate bias: 0 V
In Mol : 0.1 M
Gate bias: 0 V
In Mol : 0.125 M
Gate bias: 0 V
In Mol : 0.15 M
Gate bias: 0 V
In Mol : 0.2 M
Gate bias: 0 V
(c)
In Mol : 0.0125 M
Gate bias: − 20 V
In Mol : 0.025 M
Gate bias: − 20 V
In Mol : 0.05 M
Gate bias: − 20 V
In Mol : 0.1 M
Gate bias: − 20 V
In Mol : 0.125 M
Gate bias: − 20 V
In Mol : 0.15 M
Gate bias: − 20 V
In Mol : 0.2 M
Gate bias: − 20 V
Drain current (A)
Gate voltage (V)
Init.
Initial
30 min
100 s
1 h

(Figure S1.)

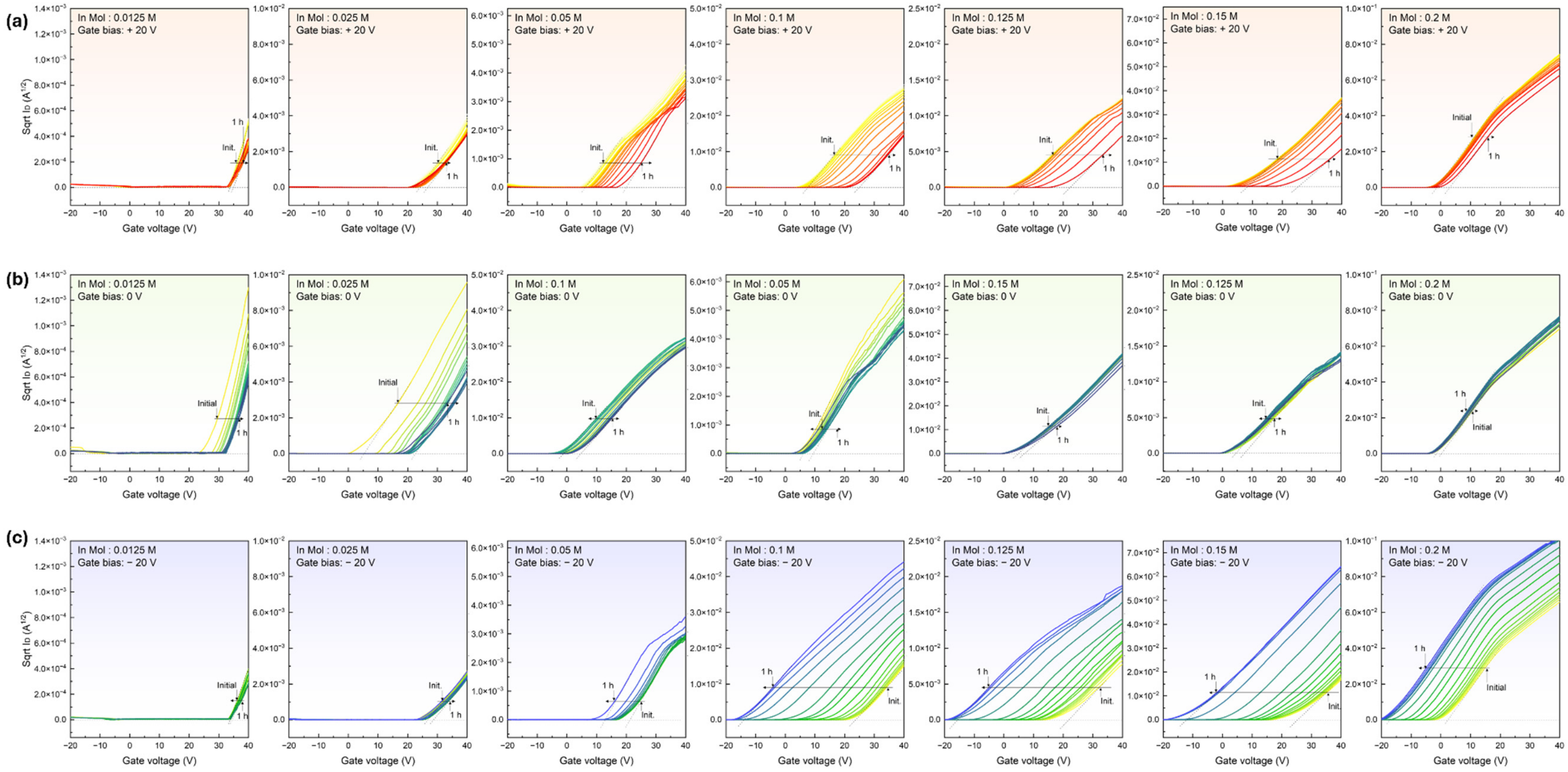
(a)
In Mol : 0.0125 M
Gate bias: + 20 V
In Mol : 0.025 M
Gate bias: + 20 V
In Mol : 0.05 M
Gate bias: + 20 V
In Mol : 0.1 M
Gate bias: + 20 V
In Mol : 0.125 M
Gate bias: + 20 V
In Mol : 0.15 M
Gate bias: + 20 V
In Mol : 0.2 M
Gate bias: + 20 V
(b)
In Mol : 0.0125 M
Gate bias: 0 V
In Mol : 0.025 M
Gate bias: 0 V
In Mol : 0.1 M
Gate bias: 0 V
In Mol : 0.05 M
Gate bias: 0 V
In Mol : 0.15 M
Gate bias: 0 V
In Mol : 0.125 M
Gate bias: 0 V
In Mol : 0.2 M
Gate bias: 0 V
(c)
In Mol : 0.0125 M
Gate bias: − 20 V
In Mol : 0.025 M
Gate bias: − 20 V
In Mol : 0.05 M
Gate bias: − 20 V
In Mol : 0.1 M
Gate bias: − 20 V
In Mol : 0.125 M
Gate bias: − 20 V
In Mol : 0.15 M
Gate bias: − 20 V
In Mol : 0.2 M
Gate bias: − 20 V
Sqrt ID (A^1/2)
Gate voltage (V)
Init.
Initial
1 h

(Figure S2.)

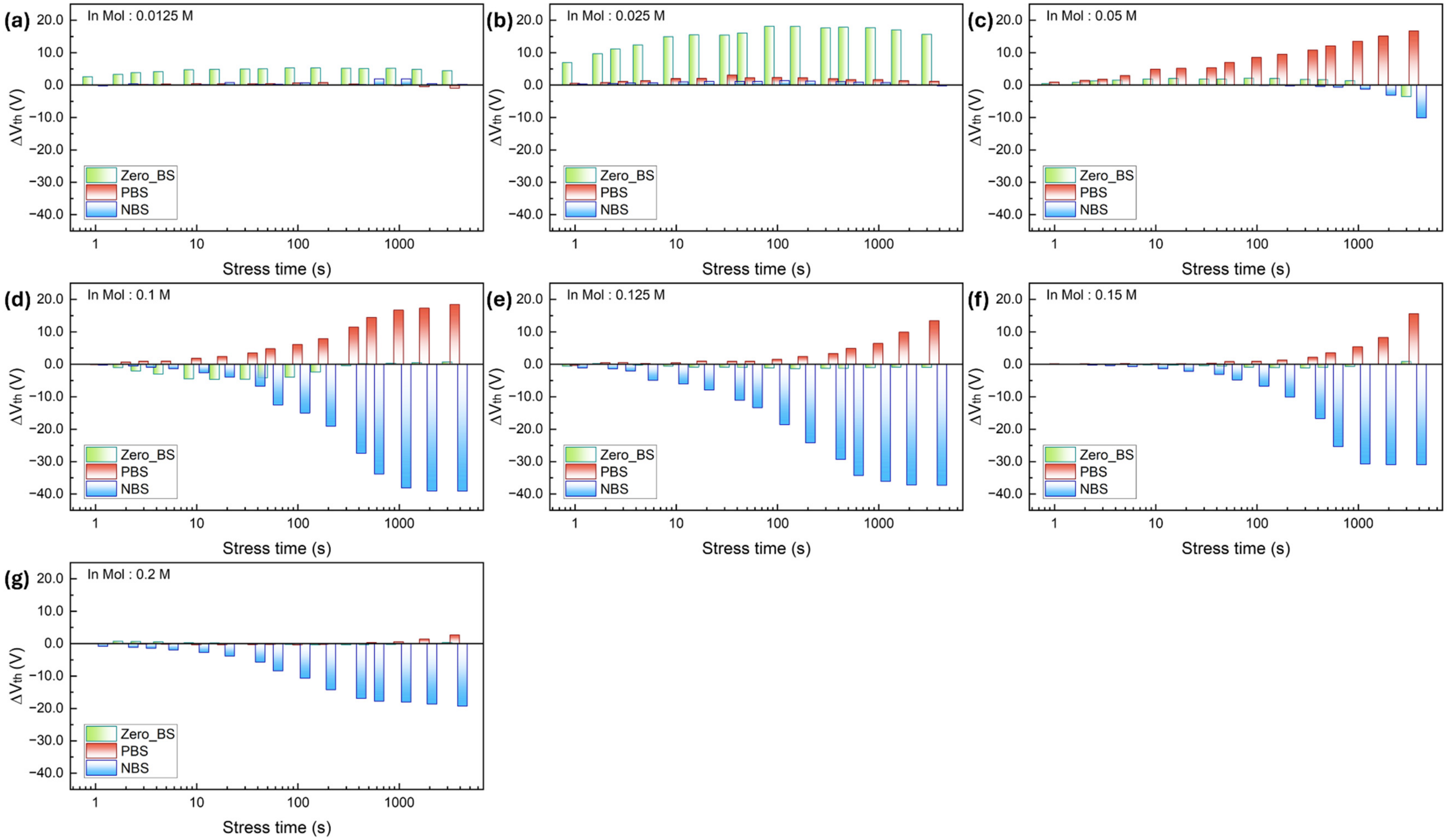
(a)
In Mol : 0.0125 M
(b)
In Mol : 0.025 M
(c)
In Mol : 0.05 M
(d)
In Mol : 0.1 M
(e)
In Mol : 0.125 M
(f)
In Mol : 0.15 M
(g)
In Mol : 0.2 M
ΔVth (V)
20.0
10.0
0.0
−10.0
−20.0
−30.0
−40.0
1
10
100
1000
Stress time (s)
Zero_BS
PBS
NBS

(Figure S3.)

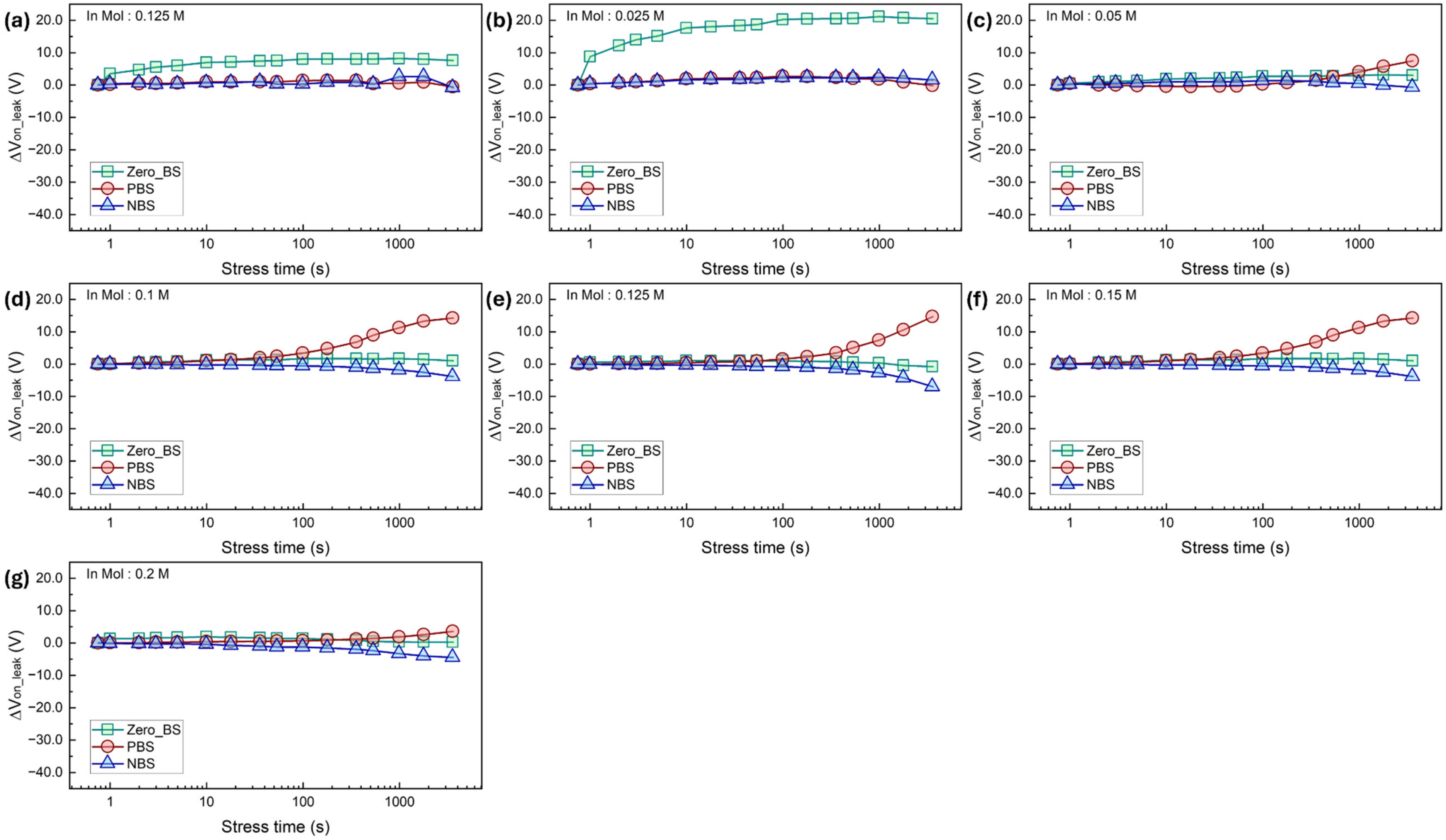
(a)
In Mol : 0.125 M
(b)
In Mol : 0.025 M
(c)
In Mol : 0.05 M
(d)
In Mol : 0.1 M
(e)
In Mol : 0.125 M
(f)
In Mol : 0.15 M
(g)
In Mol : 0.2 M
ΔVon_leak (V)
Stress time (s)
Zero_BS
PBS
NBS

(Figure S4.)

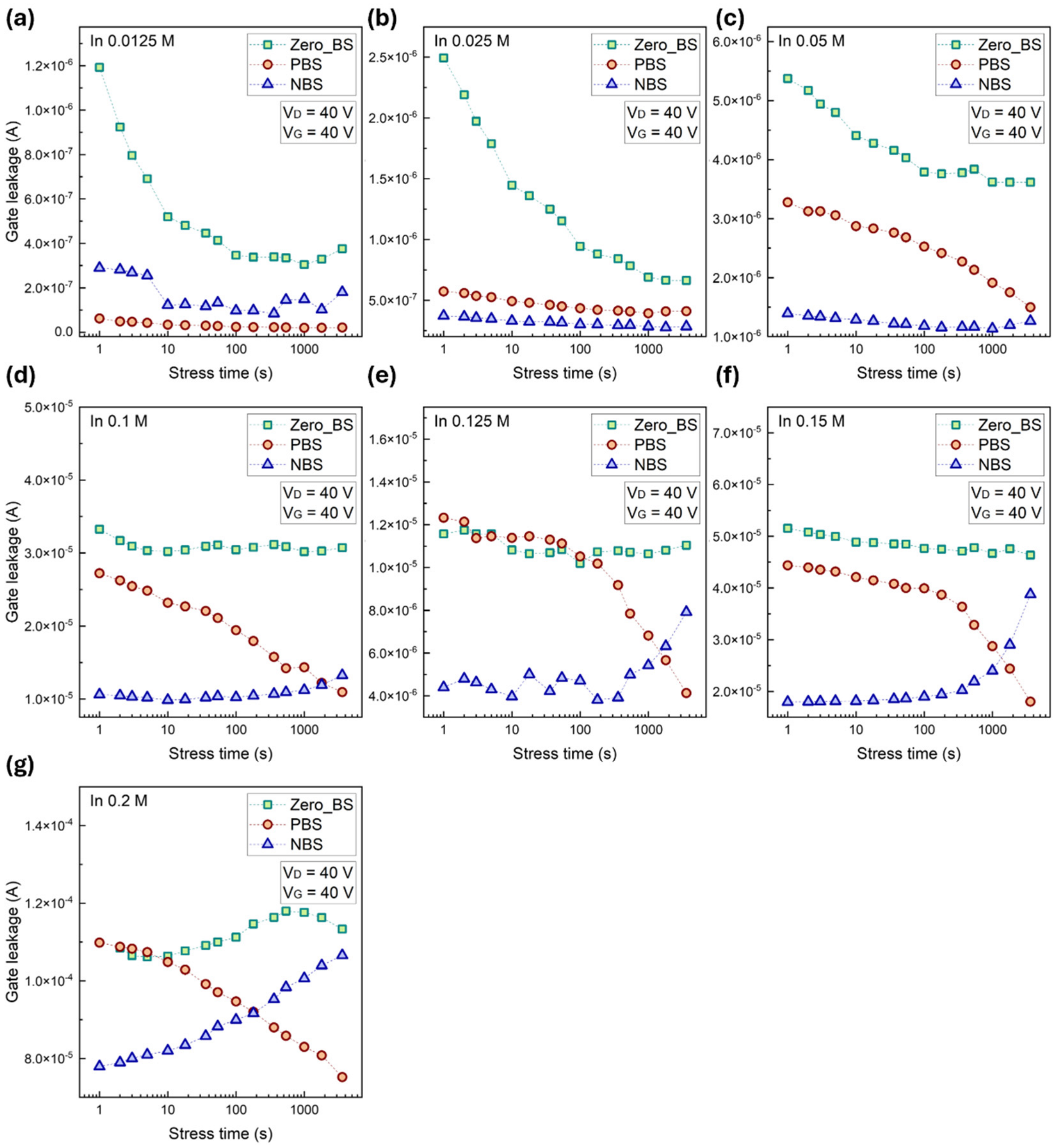
(a)
In 0.0125 M
Zero_BS
PBS
NBS
VD = 40 V
VG = 40 V
Gate leakage (A)
Stress time (s)
(b)
In 0.025 M
(c)
In 0.05 M
(d)
In 0.1 M
(e)
In 0.125 M
(f)
In 0.15 M
(g)
In 0.2 M

(Figure S5.)

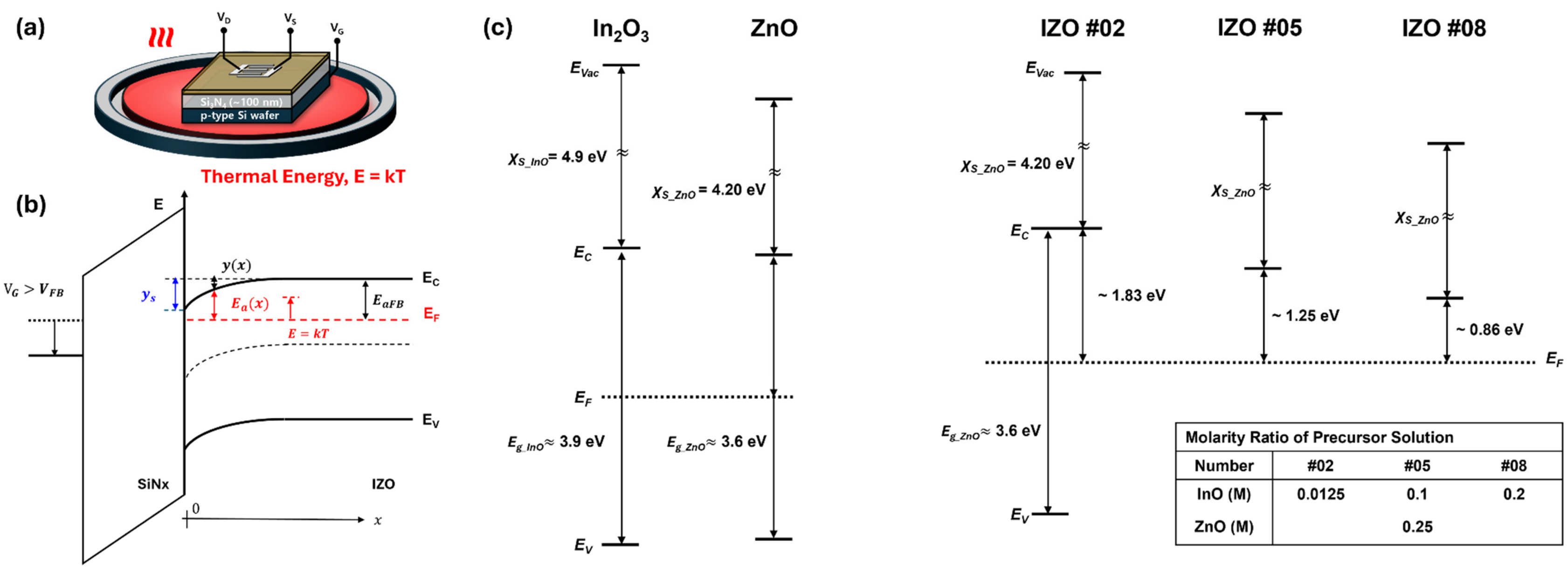
(a)
V_D
V_S
V_G
Si_3N_4 (~100 nm)
p-type Si wafer
Thermal Energy, E = kT
(b)
E
$V_G > V_{FB}$
$y(x)$
y_s
$E_a(x)$
E_{aFB}
E_C
E_F
$E = kT$
E_V
SiNx
IZO
0
x
(c)
In_2O_3
ZnO
IZO #02
IZO #05
IZO #08
E_{Vac}
X_{S_InO} = 4.9 eV
X_{S_ZnO} = 4.20 eV
E_C
E_F
E_{g_InO} ≈ 3.9 eV
E_{g_ZnO} ≈ 3.6 eV
E_V
~ 1.83 eV
~ 1.25 eV
~ 0.86 eV
X_{S_ZnO}
Molarity Ratio of Precursor Solution
Number #02 #05 #08
InO (M) 0.0125 0.1 0.2
ZnO (M) 0.25

(Figure S6.)
